# How theories of complexity and resilience affect interprofessional simulation-based education: a qualitative analysis of facilitators’ perspectives

**DOI:** 10.1186/s12909-023-04690-7

**Published:** 2023-10-02

**Authors:** Torben Nordahl Amorøe, Hans Rystedt, Lena Oxelmark, Peter Dieckmann, Paulin Andréll

**Affiliations:** 1 Department of Molecular and Clinical Medicine, Institute of Medicine, Sahlgrenska Academy, University of Gothenburg, Gothenburg, Sweden; 2 Region Västra Götaland, Sahlgrenska University Hospital, Department of Research, Education and Development, Simulation Centre West, Diagnosvägen 10, Gothenburg, SE-416 85 Sweden; 3 Institute of Health and Care Sciences, Sahlgrenska Academy, University of Gothenburg, Gothenburg, Sweden; 4 Copenhagen Academy for Medical Education and Simulation (CAMES), Center for Human Resources, Capital Region of Denmark, Herlev Hospital, Herlev, Denmark; 5 Department of Quality and Health Technology, University of Stavanger, Stavanger, Norway; 6 Department of Public Health, University of Copenhagen, Copenhagen, Denmark; 7 Region Västra Götaland, Sahlgrenska University Hospital Östra, Department of Anaesthesiology and Intensive Care Medicine/Paincenter, Gothenburg, Sweden; 8 Department of Anesthesiology and Intensive Care Medicine, Institute of Clinical Sciences, Sahlgrenska Academy, University of Gothenburg, Gothenburg, Sweden

**Keywords:** Complexity, Organizational resilience, Debriefing, Design-based research, Interprofessional education, Patient safety, Resilient health care, Safety II, Patient simulation, Team resilience

## Abstract

**Background:**

Quality of care and patient safety rely on the ability of interprofessional teams to collaborate effectively. This can be trained through interprofessional simulation-based education (IPSE). Patient safety also relies on the ability to adapt to the complexity of such situations, an ability termed resilience. Since these needs are not explicitly addressed in IPSE, the aim of this study was to explore how central concepts from complexity-theory and resilience affect IPSE, from facilitators’ perspective, when applied in debriefings.

**Methods:**

A set of central concepts in complexity-theory and resilience were introduced to facilitators on an IPSE course for nursing and medical students. In five iterations of focus groups interviews the facilitators discussed their application of these concepts by reviewing video recordings of their own debriefings. Video recordings of the interviews were subjected to coding and thematic analysis.

**Results:**

Three themes were identified. The first, *Concepts of complexity and resilience are relevant for IPSE*, points to the applicability of these concepts and to the fact that students often need to deviate from prescribed guidelines/algorithms in order to solve cases. The second theme, *Exploring complexity*, shows how uncertainty could be used as a cue to explore complexity. Further, that individual performance needs to account for the context of actions and how this may lead to certain outcomes. Moreover, it was suggested that several ways to approach a challenge can contribute to important insight in the conditions for teamwork. The third theme, *Unpacking how solutions are achieved*, turns to needs for handling the aforementioned complexity. It illustrates the importance of addressing self-criticism by highlighting how students were often able to overcome challenges and find solutions. Finally, this theme highlights how pre-defined guidelines and algorithms still work as important resources to help students in transforming perceived messiness into clarity.

**Conclusions:**

This study suggests that IPSE provides the possibility to explore complexity and highlight resilience so that such capability can be trained and improved. Further studies are needed to develop more concrete ways of using IPSE to account for complexity and developing resilience capacity and to evaluate to what extent IPSE can provide such an effect.

**Supplementary Information:**

The online version contains supplementary material available at 10.1186/s12909-023-04690-7.

## Background

Interprofessional simulation-based education (IPSE) is a means to improve patient safety, by training students and professionals in order to improve collaborative skills [1, 2]. Whilst much simulation-based education (SBE) puts the emphasis on the mastery of prescribed courses of actions [3] there is also a critique from proponents of theories of complexity science and resilient health care (RHC) that the complexity of health care is generally not considered [4–7]. From this perspective, health care is described as a complex adaptive system in which health care staff manage unpredictable variations and disturbances every day [3, 8]. Complex systems are characterized by a degree of unpredictability where situations, conditions and behaviours *emerge*, actors in the system *adapt* and *self-organize interdependently* with local perspectives and rationales that may not be obvious to other actors in the system. In such a system causality is said to be non-linear, changes in one part of the system may have surprising effects on other parts of the system. The number of relevant variables changes over time, as does the number and nature of the connections between these variables [5, 8–10]. Interprofessional health care teams are in themselves described as complex adaptive systems [11]. Such systems are said to be resilient if they have “the capacity to adapt to challenges and changes at different system levels, to maintain high quality care.” [12]. Organizational resilience emphasizes the ability to *bounce back* from adversity, the ability to find an equilibrium again, if there is an imbalance of variables and / or their connections [12, 13]. Consider, for example, the building of pop-up intensive care units in the Covid-19 pandemic to compensate for an increased need for help [14].

Erik Hollnagel suggested one way of dealing with complexity, which he called Safety-II [15]. One methodological aspect of this approach is to broaden the view on analysis and interventions, like teaching. Instead of only learning from errors, trying to prevent them from happening again, Safety-II recommends a focus on trying to understand how humans interact successfully with their social and technical environment [6]. It has also been suggested that capacity for resilience can be increased through training health care staff to manage disturbances [16]. In situ patient simulation has been proposed as a tool to examine complexity and resilience in every day clinical work [17, 18]. SBE was also suggested for the training of resilient skills in other domains, such as electricians [19]. Using SBE to train nursing students to manage complexity has been examined [20]. There is, however, limited empirical data on how theories on complexity and resilience could be incorporated into IPSE when training interprofessional health care teams, which is of central concern in this study [3, 21]. IPSE involves interprofessional teams managing medical challenges in full-scale simulation scenarios followed by post-simulation debriefings. The debriefing is seen as essential for participants to learn from the scenarios and is led by a facilitator [22]. Further, it has been argued that simulations and debriefings have the potential to explore the complexity and emergent nature of everyday problem-solving [23–25].

Although SBE may have paid some attention to complexity by exploring variations and participants’ perspectives [26], and even though it is a widespread praxis to highlight (but not necessarily to analyse) what went well in a scenario, the main focus is often put on training the right course of actions by adhering to particular sets of prescribed recommendations, guidelines or algorithms [3]. A major criticism of this approach is that when the complexity that is found in everyday clinical work is not taken into consideration there is a risk of favouring imagined challenges and simplistic learnings which are not relevant in reality [18, 23, 27–31]. An objection is that courses with this approach might be effective in teaching pre-defined learning goals but might fail to help participants to apply the newly acquired skills and might be too rigid to be applied in different contexts. Following this line of argumentation, it has been suggested that SBE, in helping health care professionals to treat patients effectively and safely, needs to balance two dimensions [32–34]. The first dimension, termed effectiveness, concerns how closely and precisely SBE helps to achieve pre-defined learning goals. The focus of the other dimension, labelled the innovative, is to help learners to adapt what they learned to different contexts. One might assume that a focus on the first dimension only might lead participants to be locked in the learning situation at hand such that they might not be able to apply what they have learned in new situations. Conversely, a one-sided focus on the second dimension may allow learners to be very flexible and to juggle new ideas but might leave them lacking the background knowledge and practice opportunities to actually apply this new knowledge. Addressing both dimensions would help participants to acquire the state of the art in a certain field and to be able to apply the related knowledge, skills, and attitudes in different contexts - building adaptive expertise. The latter aspect is also described as building resilience capacity [35]. To address these issues, the present study explores the possibilities of designing debriefing principles in IPSE that not only focusses on pre-defined learning goals in terms of prescribed guidelines and algorithms, but also contributes to improving patient safety by accounting for the complexities of teamwork and the need for resilience capacities in emergency care. Further, a central assumption in this study is that complexity and resilience perspectives could be useful tools for developing such debriefing principles. A first important step to explore this assumption is to gain knowledge of the relevance of these theoretical perspectives and, if relevant, how they could be incorporated into IPSE practice. Thus, the aim of this study was to explore how central concepts of complexity and resilience could affect IPSE from a facilitator perspective.

## Methods

### Study design

This study was part of a research project which used a design-based research methodology [36, 37] to explore and develop principles for debriefing which incorporate concepts from complexity theory and the field of “resilient health care”. This qualitative study used thematic analysis [38] on transcripts from focus group interviews with facilitators on post-simulation debriefing in IPSE.

### Setting of the study

Pre-graduate nursing and medical students at the University of Gothenburg participated in a course called “Interprofessional care of the acutely ill patient – team training in simulated environments” in their final term. The course was mandatory and was provided at the Simulation Centre at Sahlgrenska University Hospital. The purpose of the course was that: “The student should improve his or her skills in communication and collaboration with other health care professions in order to be able to take care of an acutely ill patient in an effective and patient-safe manner”. The overall goal of the course was to develop interprofessional and teamwork skills applying the principles of Crew Resource Management (CRM) [39].

**Fig. 1:**
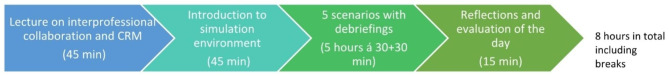
Program for 1-day interprofessional simulation-based education course

A course day started with an interactive lecture on principles for effective teamwork, leadership and communication (CRM) followed by an introduction to the simulation environment. After the introduction, the students took part in five different scenarios, either as actively involved in the simulated care team or as observers. Each scenario was then followed by an immediate facilitator-led post-simulation debriefing. The course ended with reflections and an evaluation (Fig. 1).

Special emphasis was put on collaboration, communication and learning about each other’s professional roles, responsibilities, and perspectives. Other focus areas were: using the ABCDE (Airway, Breathing, Circulation, Disability, Environment) algorithm to systematically approach patients, raising critical issues through “speak up”, gaining and keeping the overview of the development of care, team alignment, avoiding fixation through explicit re-evaluation/summation, and finally distribution of workload. The scenarios were set in a simulated medical ward, a surgical ward, and at a primary health care centre. All represented common workplaces for junior physicians and nurses. Simulated medical conditions were common acute conditions i.e. chronic obstructive pulmonary disease exacerbation, stupor due to hypoglycaemia, confusion caused by postoperative bleeding, postoperative sepsis, and ketoacidosis. Most patients also had comorbidities such as hypertension or atrial flutter. All scenarios were carefully designed to incorporate both medical-technical, nursing and CRM aspects at different times and to make sure both professions had specific tasks and challenges. The scenarios varied in terms of severity, onset of problems, presence of relatives, and availability of help. The debriefing followed Steinwachs 3-phase model of description-analysis-application [40]. Approximately 200 students each semester attended in groups of 8, with 4 student nurses and 4 medical students in each group. While 3–5 students were active in each scenario, their peers observed a live video stream in an adjacent room. All student groups were facilitated interprofessionally by one nurse and one physician.

### Sampling

The participants were facilitators conducting debriefing in the IPSE course. All of the 20 facilitators teaching on the course were invited to participate in the study, 9 agreed to participate. The facilitators were 44 years old on average (range 32–61), with 7 women and 2 men. Six facilitators were specialist doctors and three were specialist nurses. The facilitators had been facilitators for 4.5 years on average (range 2–8 years). During this research project the facilitators facilitated 22 scenarios on average (range 8–35) on the IPSE course. All facilitators had participated in one or more simulation instructor courses of at least three days duration. All the facilitators were assigned to one focus group which were interviewed multiple times.

### Data collection

**Fig. 2:**
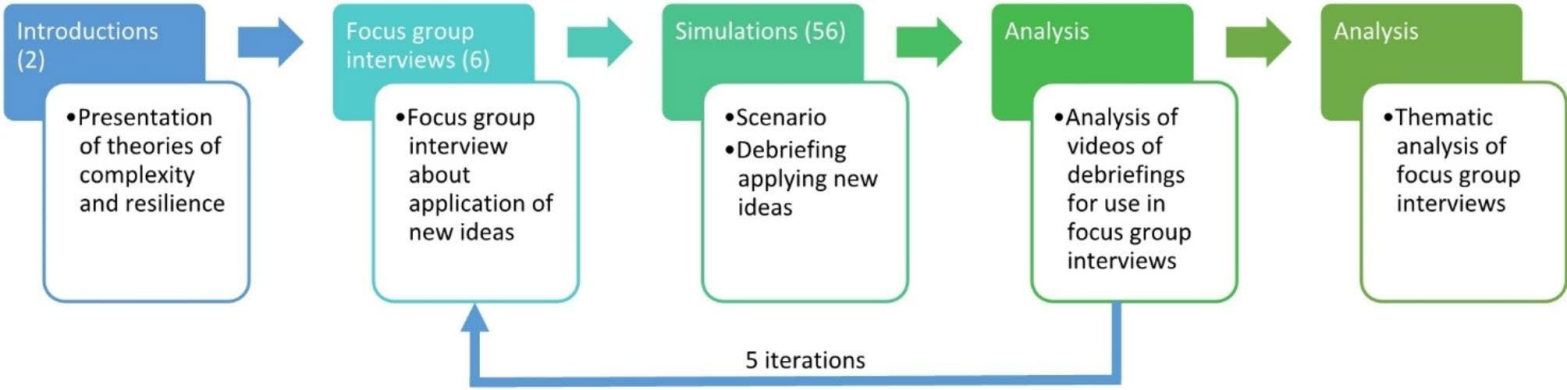
Data collection and Analysis process

Data was collected from August 2017 to June 2018. Data collection took place in an iterative process (Fig. 2).

1. Fifty-six video recordings of debriefings on the IPSE course (approximately 17 h).
2. Six video recordings of focus group interviews (approximately 11 h).

The primary data included video recordings of the six focus group interviews which were conducted by first author, a PhD-student, specialist physician in anaesthesiology and intensive care and experienced facilitator. While recording audio was the main objective, the video format was chosen, to identify each speaker and to secure identification of which debriefing clip was shown and discussed. Observational notes were taken by one co-author interchangeably as a support for the following analysis. The first two focus group interviews followed an introductory session where the central concepts of complexity theory and RHC were presented. Semi-structured interview guides covered questions on how the facilitators applied their growing understanding of complexity and resilience in their debriefings on the IPSE course (Supplementary Material 1). This double function of the focus group interviews as data collection and development of the intervention complies with the principles of design-based research. In total 56 debriefings were videotaped, and each video recording was reviewed and parts of the videos where facilitators asked questions and discussed subjects of interest for the research question were identified and transcribed. These sections were labelled and presented in the following focus group interview as basis for further discussion about the understanding and application of complexity theory and resilient health care in IPSE.

### Data analysis

Debriefings were transcribed and analysed as a tool to identify situations to show and discuss in the following focus group interviews. This generated 101 codes. When all video recordings of the focus group interviews were collected, the interviews were transcribed verbatim by the first author. All video recordings of focus group interviews were analysed inductively using thematic analysis following 6 steps according to Braun and Clarke [38]. Microsoft Excel and MindMup 2 for Google Drive were used to organize and structure the data. All authors familiarized themselves with the data by reading the transcriptions and taking note of initial ideas. Then, sections of the data where facilitators discussed complexity and resilience were identified. These sections of the transcriptions underwent semantic coding creating 115 short codes and a second recoding according to Saldaña [41], creating 246 more descriptive codes by first author. Initial themes were selected and discussed by all authors. The dataset, codes and initial themes were further reviewed and mapped using mind maps and diagrams in several iterations to answer the research questions until consensus was reached among the authors. In addition, preliminary ideas were presented and discussed in a multi-professional workshop with the participation of three well-established academic Swedish simulation centres, comprising 11 participants which generated ideas for the analysis and theme development. Each of the produced and finalized themes were described and put into a narrative.

## Results

**Fig. 3:**
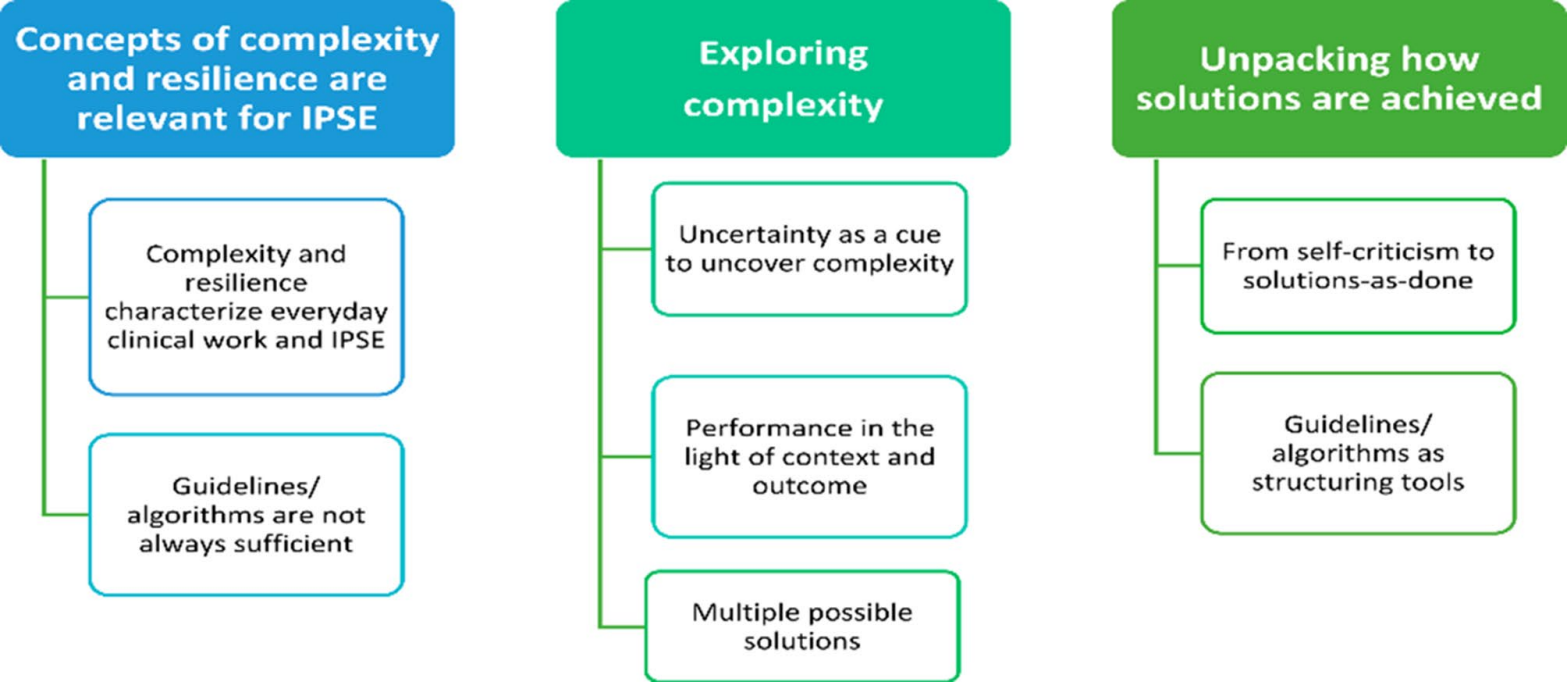
Themes and Subthemes

Three themes and seven subthemes were identified (Fig. 3).

### Concepts of complexity and resilience are relevant for IPSE

From the very start the facilitators found the ideas about complexity theory and resilience relevant and important to work with. They described examples from everyday clinical work as clinicians and in their work as facilitators in IPSE. The first sub-theme shows how these ideas are seen as relevant for both everyday clinical work and IPSE. This stance is further reinforced by the second sub-theme, describing situations in which guidelines/algorithms not always sufficient for action.

#### Complexity and resilience characterize everyday clinical work and IPSE

Facilitators described complexity and the need for resilience as regular features of everyday clinical work, being often messy and uncertain. Thinking of highly dynamic situations, it was described how professionals at times struggle to manage seemingly chaotic situations, to find structure and solutions, not always adhering fully to pre-existing guidelines. Although the facilitators acknowledged that the scenarios were not specifically designed with complexity and resilience in mind, they were surprised how much the scenarios still offered in those dimensions and how closely they evoke issues close to the actual clinical work. According to the facilitators, a common word for complexity used by students was “messiness”, often referring to situations in which “many things happen” with “many people involved”.

The facilitators found examples of complexity and resilience when something unexpected seemed to cause uncertainty and initially substandard behaviour. For example, a student seemed to fail to communicate effectively, initially not directing the information to any team member specifically, but sorted it out a little later. In another instance a student criticized himself for getting stuck in the scenario but also noted how he overcame this when he focused on the tasks given by the team leader, thus demonstrating the resilient capacity of team and individual. Moreover, the facilitators repeatedly referred to the complexity and resilience of everyday clinical work and pointed to the need for the students to learn about and handle this in IPSE.

#### Guidelines/algorithms are not always sufficient

The facilitators talked about how recommendations or guidelines for managing acute situations (e.g. ABCDE and CRM) do not always fit the situation, resulting in a need to either act spontaneously or to deviate from existing guidelines to optimize patient treatment. A facilitator described an example involving a medical student who is alone with a vomiting patient in the scenario and who does not know how to proceed. The facilitator interpreted this as follows:

*“It’s such a fantastic expression for ´I have no algorithm to enter the room, where someone has vomited. What am I doing now? What are possible diagnoses? What is expected of me?´” (F3)*.

With the perspective of complexity and resilience in mind the facilitators raised their concern. They acknowledged the need for repeatedly practicing “the right course of action according to specific recommendations and guidelines”. However, they also expressed concern that focusing solely on such algorithmic approaches could create expectations among the students (and facilitators for that matter) that such algorithms would always be in place and should always be followed no matter what. Such rigidity, they argued, was not suitable for the complexity of actual care. It was argued that this may hamper the necessary creativity when adaptations are needed. According to facilitators this also risked creating conflict, when clinicians perceived that colleagues “violated” recommendations. When adhesion to the algorithm is the only concern, the mere deviation becomes problematic, and the – possibly good - reasons for the deviation are lost from sight. An example was the facilitators’ discussion of a video clip where a student nurse referred to her earlier ABCDE training and objected to deviations by a medical student who, in order to gain time, deliberately did not follow ABCDE strictly, taking shortcuts.

According to facilitators such “fixation” on recommendations and guidelines deprives students of valuable learning opportunities, when it comes to taking the initiative when things do not go as expected, especially when colleagues do not do as expected.

Initially, concern about adaptation and allowing deviation was also raised:

*Well, but somehow you can argue against this idea… without protocols, then, if it was not actually written at all, that we have to drive on the right side of the road, what would the roads be like then? (F2)*

However, there was agreement that being too focused on guidelines and recommendations by fixating only on minimizing performance gaps was problematic as it ran the risk of oversimplification, not taking the complexity into account and dismissing examples of resilient behaviours as creatively solving a challenge to success.

### Exploring complexity

As the facilitators experimented with applying the concepts of complexity and resilience in their debriefings, specifically, they found several common activities in debriefing that could be understood in new ways.

#### Uncertainty as a cue to uncover complexity

The facilitators discussed uncertainty among the students as a central feature of complex situations. A facilitator recounted an example with a medical student. In the scenario she became confused about the next steps, as the nursing students had already carried out the examinations she had planned before entering the room. The student was confused, because she was taught how to act, when being part of the scene from the beginning, but not when arriving in the middle of a case. The facilitators discussed how these kinds of small challenges that were unforeseen by the students often gave rise to feelings described as awkward or stupid. However, the facilitators reported that they suspected that the signs of uncertainty and hesitation also had to do with the unpredictability of the complex situation per se rather than incompetence, as expressed here:

*… there may be uncertainty about many things, and I think that usually some kind of complexity is involved, while it is only occasionally purely about lack of knowledge, that one does not know (F3)*.

It was further reasoned by the facilitators that since adept professionals seems to experience uncertainty and messiness quite often, this should be seen as something common and normal.

The unexpected ambiguous moments and the feelings they give rise to needed to be attended to, according to the facilitators, the student could not be left with a sense that they should/could have done something different to avoid this. They argued that the aim should be to learn to work/train while experiencing effects of complexity, rather than aiming to get rid of this experience, because such situations will always arise. The facilitators argued that experience of uncertainty should not be seen as a flaw, either by the student or by the facilitator but rather should be understood as a cue to explore the situation, to use this feeling as a springboard to ask what was needed in the situation, not only as a question in the debriefing, but as a proxy to develop strategies for the students to be used in the next scenario and ultimately in clinical practice.

#### Performance in the light of context and outcome

The facilitators discussed how there seemed to be a tendency, albeit uneven, among the facilitators to focus on an individual’s performance gap as opposed to seeing the team perform as a whole. In a discussion about an example where a student nurse was overloaded with tasks and found it difficult to communicate this, a facilitator said:

*Because I mean, it’s not ONE person, when you end up in that situation, so it’s not the individual, it’s the group, it is a collective thing (F9)*.

The facilitators found that we cannot ignore the fact that individuals do not act in a vacuum but continuously react to the dynamics of the situation, new information and their teammates. According to the facilitators it would often be too simple to only see individual gaps without taking the context into consideration.

Facilitators noted that the focus of performance examination was often put on the degree of adherence to guidelines and not how this affected outcome for the patient:

*“…but a little bit like this, also in the debriefings, there is a lot of focus on how tasks are performed, and so on, but actually…[] How did it actually turn out for the patient? That is what the objective is. The objective is not to fulfil all these performances” (F6)*.

Facilitators did not deem it wrong to look at performance gaps, but they did, however, maintain that this focus at times became detached from the outcome. Facilitators recalled that they themselves contributed to this focus on performance gaps by making it the specific, explicit learning objective of a scenario. To that extent they, at times, forgot to acknowledge that the patient’s condition had actually improved by the end of the scenario. Thus, according to the facilitators, the focus should not always be on whether things were performed in accordance with prescribed guidelines, but rather on how the things done contributed to the successful resolution of the case.

#### Multiple possible solutions

When exploring how to deal with complexity the facilitators returned to the notion that exploring multiple possible solutions was important. For example, this related to a medical student being passive while a nurse student was overloaded:

*And what I think is the most ingenious thing here is not to go for solutions there, which I think is “linear”, but to embrace this complexity….When it’s messy, how can you make it a little less messy? And that it is not about finding […] ONE reason and ONE solution […] and that […] you have multiple possible alternatives (F3)*.

The idea was, in order to acknowledge the complexity of the situation, to be exploring advantages and disadvantages in different suggested solutions instead of finding one single course of action:

*No, but then I think that we wouldn’t need to simulate, if you simply are to deliver truths. The idea is that someone should feel it themselves, otherwise we could have skipped the debriefing, and just sat and told them truths (F9)*.

According to the facilitators exploring several solutions with their features was about improving the ability of participants to make decisions in unfamiliar situations and to develop feasible decision and action strategies.

### Unpacking how solutions are achieved

A consequence of seeing team performance in the light of complexity and resilience was an increasing interest in the need to place more attention during debriefings on how participants overcame and solved the challenges they encountered.

#### From self-criticism to solutions-as-done

The facilitators found that self-criticism seemed to stand in the way of seeing. They agreed it was common that students were self-critical and that this was most commonly about the situation being perceived as messy or oneself not being calm, not being in control. One facilitator expressed it like this:

*It seems like there are quite a few people who are very self-critical and quite hard on themselves and easily find things they did wrong, but who may have a harder time finding things that they did well (F5)*.

The facilitators reflected upon several reasons for being self-critical: pre-emptively expressing self-criticism before receiving criticism in order to save face, lack of experience, a kind of black-out i.e. having difficulty remembering what went on in the scenario, especially what went well.

Facilitators generalized that it seemed like many students have high expectations towards their own performance, that students expect of themselves that they should be able to act according to an imagined ideal performance, and that they should be in control from start to end of the scenario, understanding everything with clarity from the get-go. The facilitators contrasted this to the work of the experienced professionals, who may not always have this kind of control from the beginning, nor the expectation that this should be the case.

The facilitators also pointed out that students often neglected the fact that after the things that did not go perfectly, many things were actually solved. One facilitator pointed out “it is so great” when you highlight for the students “you had a challenge, and you DID get it in the end”. Thus, the team’s and the individual students’ resilience was demonstrated, which was regarded as being important.

#### Guidelines/algorithms as structuring tools

One facilitator highlighted how the learning goal cannot merely be to acknowledge that situations can be messy. “`You just have to adapt to the situation` is quite an unsatisfactory conclusion after a simulation” she said. The facilitators agreed that the role of facilitators and the simulation was to help participants to acknowledge complexity *and* to help them with a transition from messiness to clarity and to implement the best possible care for the patient under the given circumstances. A facilitator compared scenarios to clinical work and described a similar dynamic moving from initial messiness to achieving an order that helps in managing the case.

So, while there was agreement on the idea that exploring complexity and resilience was important, not considering prescribed guidelines/algorithms (i.e. structure) would be unreasonable:

*Although I’m still thinking somewhere that this is going to be really weird, because the structure is there to help, to get to know the working methods and the structure, and they can hang on to this. But sometimes it does not work and that’s fine, but they still have this structure to hang on to (F9)*.

Thus, the facilitators argued that the course learning goals such as mastering guidelines and recommendations as ABCDE and CRM are still extremely relevant and may very much constitute the basis for developing resilient behaviours.

## Discussion

Facilitators find that central concepts from complexity theory and organizational resilience are relevant to teach and train in IPSE. In order for students to increase their capacity for resilience, debriefings should not only focus on corrections related to prescribed guidelines and algorithms but also on making students aware of the complexity of the situations that they encountered in the scenarios, how they succeeded and what they did to overcome such complex challenges.

This study shows how addressing complexity and the need for resilience could prepare students to understand and act in the complex nature of actual health care [16]. IPSE offers a lot of potential for this type of training, since scenarios can incorporate unpredictability, emergence, ambiguity, disturbance, uncertainty, many things happening with many people, which are the characteristic features of theories on complexity and resilience [3, 20, 23, 30]. In accordance with several studies our findings indicate that simulation can be used to address participants’ adaptive capacity or adaptive coordination, i.e., that resilience and that there is a need for this [3, 42, 43]. This study also stresses the need for facilitators to guide students in this endeavour.

Our findings suggests that when learning to handle complexity, guidelines and algorithms may at times not be helpful. However, the facilitators also stressed seemingly contradictorily that guidelines and algorithms serve as strategies to handle complexity, becoming tools for resilience. The two sub-themes “Guidelines/algorithms are not always sufficient” and “Guidelines/algorithms as structuring tools” reflect this apparent inconsistency.

This study raises concern that SBE often puts a one-sided focus on what Schwartz et al. refer to as the effective dimension of teaching, emphasizing training students to follow pre-defined courses of actions and avoid deviations [3, 34]. This is exemplified in cardio-pulmonary resuscitation-training (CPR) and expressed clearly in deliberate practice and mastery learning in controlled simulated environments [44–47]. Several studies have pointed out that while such training is important part of skills acquisition, only training such methods may not convey, prepare, teach or train students for the complexity of health care work and the needs for teams to adapt to its ever-changing nature, thus stressing the importance of incorporating the innovative dimension of teaching [18, 23, 28, 48, 49]. The difference in these approaches corresponds to the difference between looking at how “work is done” in everyday clinical life and looking at how work should be done or “work-as-imagined” [3, 18]. The facilitators in our study seem to be saying that simulation unfolds its potential better, if it relates as closely as possible to the actual challenges of “work-as-done”.

The facilitators emphasized that referring to and using prescribed guidelines and algorithms in IPSE is still extremely important. It is necessary to scrutinize how guidelines and algorithms were used as strategies to solve cases and bounce back from challenges, not forgetting that guidelines and algorithms as CRM principles often are also designed to deal with complexity [50]. However, it was suggested that if this is done after exploration of perceptions, disturbances, uncertainties, adaptations etc., it would lead to more realistic, feasible learning points i.e. addressing both the effective and the innovative dimensions [49].

Our study indicates that uncertainty may not necessarily mirror an individual’s incompetence but may be seen as a perception of the complexity of the situation, with its implicit uncertainties [20]. Starcke et al. describe decision situations on different levels of uncertainty, related to the level of information available [51]. The less information there is, the more interpretations are possible, the fewer hints there are to assess those interpretations, and the higher, therefore, the complexity. Situations and corresponding experiences become less and less uncertain as more information is acquired [49]. Uncertainty can stem from both an inadequate understanding by the individual(s) and / or an incomplete information base in the situation as such [52]. This line of argumentation supports our findings in which the facilitators argued that students mostly interpret their own uncertainty and experience of messiness as rooted in their own lack of skills, frequently blaming themselves for their perceived inadequacy. This would entail supplementing discussions of what the ideal solution of the case would be and what the individual can improve to reach it with discussions that emphasize the value of “muddling through”, working towards local and temporal optima [3, 18, 53, 54].

In order to increase the capacity to act resiliently, exploring not only the individual student’s actions in isolation, but also exploring team and context and why overall outcome turned out the way it did was found to be important. This is in line with Fenwick and Dahlgren’s call for the use of simulation to train students to become more “attuned” to the situation, to be aware of the emergence and messiness of ambiguous elements in the scenario and to highlight these as important learning opportunities in debriefing [23].

Our study emphasizes the need to take a closer look at why things went well in the scenario as part of exploring complexity and highlighting resilience. Pointing out resilience is important as students often cannot see their own successes or overcoming of challenges. Rudolph et al. acknowledge the effort and intention of participants to do right and recommend digging deeper to explore participants’ perspectives [55]. However, while this approach has the potential to explore complexity, the aim is to identify participants’ knowledge gaps that can explain their performance gaps so that they can be corrected, which means that it is mainly concerned with Schwartz’ effective dimension [34]. Several authors state that while it is common practice to ask for what went well, focus is still on what went wrong. They find that participants often do not really know why things went well [3, 30], therefor Dieckmann et al. propose an addition to the traditional “corrective approach” to simulation by exploring how good performance is produced [3].

The study set out to explore the effect of introducing concepts from organizational resilience as into IPSE, meaning the focus was on the team as a whole, seeing the team as a complex adaptive system in itself [11]. Hence, the focus was initially on team resilience excluding individual or psychological resilience [56]. However, the facilitators were also concerned with the students’ well-being, their emotional reaction, how they felt awkward or uncertain and how they were overly self-critical, blaming themselves. This attentiveness is in accordance with the intention of providing the students with a good learning experience in a psychologically safe learning environment, which is considered a key factor in successful IPSE learning [57, 58]. Some definitions of team resilience take both the function of a team and the well-being of the individuals into account [59]. Thus, our findings point to the need not to focus solely on the team/organizational level leaving out individual level when exploring complexity and resilience, but rather to address both.

### Study strengths and limitations

Within the simulation community there is a call for theory-driven research on SBE [60, 61] and within the community of resilient health care there is a call for empirical studies attempting to explore the practical applications of the theories [7, 62]. A strength is that this study meets these calls.

Trustworthiness and dependability were sought in line with Lincoln and Guba [63, 64]. To secure credibility questions in focus groups interviews were reviewed by all authors and follow up questions allowed exploring a topic in detail and could be revisited in a later focus group interview. The authors obtained a good familiarity with the focus group participants as authors and participants met many times over the course of the project (both on course days and in focus group meetings), thus establishing prolonged engagement and persistent observation [63, 65]. During the data analysis the first author continuously critically examined that the coding and video-clip examples that were relevant for the research question. Counter examples to the researcher’s preconceived ideas were deliberately sought. Results and ideas were thoroughly examined and discussed by all the authors and preliminary findings were discussed with collaborating researchers from other Swedish simulations centres. The authors of this work comprised of both male and female researchers within nursing, medicine, and the learning sciences with various professional backgrounds.

To increase the potential for transferability [63] and diversity in answers a variation of facilitators was sought. Some facilitators were very experienced and some less (none were beginners), including both nurses and physicians which contributed to multiple interprofessional perspectives. Also, the facilitators were associated with two different simulation centres in Gothenburg (University and Hospital affiliated) which might benefit diversity. Gathering data from other centres in Sweden may have been beneficial for more diversity in answers. It can be seen as a limitation that of 20 invited facilitators only nine chose to participate and that one facilitator only participated in the first round of the focus group interview. Many of the invited facilitators expressed interest but claimed time constraints, all facilitators did not participate in every focus group interview (but did participate in testing newly conceived ideas in debriefings). This lack of consistency may contribute to less variate perspectives. The number of focus group interviews was limited to six as further reflections on concepts introduced did not reveal new insights. The reporting of this study was guided by consolidated criteria for reporting qualitative research (COREQ) [66].

### Implications for IPSE

By receiving training about building awareness of complexity and about the need for resilience in IPSE, the students might gain more relevant strategies to handle the complexity of teamwork. In this way the findings also contribute to further understanding how the concepts of complexity theory and resilience can inform IPSE practices for the sake of improving the quality of health care. In addition, a possible learning outcome could be to encourage students to discuss this in real life practice, thereby building resilience.

## Conclusions

This study suggests that IPSE provides the possibility of exploring complexity and of highlighting resilience so that capability in these areas can be trained and improved. To teach and train in IPSE and in order for students to increase their capacity for resilience, debriefings should not solely focus on corrections related to prescribed guidelines and algorithms but should also be used to make students aware of the complexity of the situations that they found themselves in during the scenarios, how they succeeded and what they did to overcome such complex situations. Further research is required to deepen our understanding of how these findings can be applied and evaluated more concretely in debriefings and to increase our knowledge of how this might contribute to the professional development of the students and ultimately to increased patient safety.

### Electronic supplementary material

Below is the link to the electronic supplementary material.

Supplementary Material 1

## Data Availability

The datasets used and/or analysed during the current study are available from the corresponding author on reasonable request.

## Declarations

All research participants (facilitators) and course participants (students) received written and verbal information about the study before giving informed oral and written consent to participate in the study.

Participants were informed that participation was voluntary, and that they could withdraw at any time without any consequences.

## Abbreviations

IPSE: Interprofessional simulation-based education
SBE: Simulation-based education
RHC: Resilient Health Care
CRM: Crew/Crisis Resource Management
ABCDE: Airway, Breathing, Circulation, Disability, Environment
F#: Facilitator number
COREQ: Consolidated criteria for reporting qualitative research
