# Supplementary material for: How theories of complexity and resilience affect interprofessional simulation-based education: a qualitative analysis of facilitators’ perspectives
Source: BMC Med Educ. 2023 Oct 2;23:717. doi: 10.1186/s12909-023-04690-7 (PMC10546720; doi:10.1186/s12909-023-04690-7)
Supplement: Supplementary file 1 — Supplementary Material 1 [file 12909_2023_4690_MOESM1_ESM.pdf]

Focus Group Guides

Focus Group Guide Meetings 1 and 2

A. PURPOSE OF THE INTERVIEW

B. AGENDA

C. OPENING QUESTION

1. What challenges do you face in your debriefings?

D. IN-DEPTH QUESTIONS

2. Do you have any concerns about how deeply the students reflect in the debriefings?
3. What is your impression of the students' self-criticism in the debriefings?
4. Do you have any thoughts on whether students develop observable interprofessional behaviours in the scenarios during the day?
5. What do you think about using video in debriefing?

E. PRESENTATION OF IDEAS AND THEORIES

6. What are your immediate thoughts on these ideas?
7. How are these ideas relevant to simulation and debriefing?

F. DEVELOPMENT OF VIDEO-ASSISTED DEBRIEFING METHOD FOCUSING ON STRENGTHS AND WHAT WORKS

8. How could you put this into practice in a very concrete way?
9. What 3-5 points would you like to bring to your next debriefings? (Action plan[1])

G. CONCLUDING QUESTIONS

H. SUMMING UP

10. Would anyone like to add something?

---

[1] The ”Action plan” consists of specific points in the debriefing script that are to be tested in upcoming debriefings e.g. new questions

Focus Group Guide Meeting 3

A. WELCOME

B. RECAPITULATE

C. OPENING QUESTION

1. What are your impressions and thoughts after the debriefings you have held?

D. IN-DEPTH QUESTIONS

2. In the debriefing videos 4 topics stood out:
    1. How to get an overview of topics to debrief and identifying the most important ones (some of you have tested plus/delta)
    2. Exploring what worked well or what students succeeded in
    3. Deepening and concretizing what was challenging
    4. Concretizing lessons learned in the application phase

Each video clip is shown with a short introduction about the topics

The facilitator in question is first given the opportunity to respond to

1. What were you thinking here?
2. What did you want to achieve here?
3. Was there anything you think you succeeded in?
4. Were there any challenges?
5. How did you overcome these challenges, what were your strategies?

3. Topics from the first two meetings:
    1. How to use learning objectives more clearly
    2. Understanding of what you mean by interprofessional competences. Is it synonymous with CRM? What do you think?
    3. Introducing concepts of complexity and resilient behaviour for students in a lecture. Should this be done? How?

E. INFORMATION REGARDING CONCERNS ABOUT THE PROJECT'S POTENTIAL EFFECT ON EXAM

Some students have expressed a wish for more personal feedback.

4. Is there a risk of losing something, when you direct questions more to the whole group instead of individually?

F. ACTION PLAN ADJUSTMENT

5. How can you translate these discussions in a very concrete way into adjusting the current action plan?
6. What does our new action plan look like?

The group creates a new joint action plan (3-6 points)

G. SUMMING UP

Focus Group Guide Meeting 4

A. WELCOME

B. AGENDA

C. OPENING QUESTION

1. Are there are any thoughts or spontaneous reflections that you have been considering since the last time?

D. IN-DEPTH QUESTIONS

<u>Application phase</u>

2. Earlier you discussed "concretization in the application phase" – I will show video clips where "time to think" is given and you use the whiteboard
3. These clips also relate to how the word "strategy" is used which you discussed earlier

<u>Intended learning objectives</u>

4. Earlier you discussed "learning objectives" and I want you to discuss the use of the "learning objectives" and look at how the whiteboard is used in this regard.

<u>Solution focus (or "what works")</u>

5. One of the objectives was to work more with "what works", and despite perceived challenges get the students to see their strengths and create positive target images. I would like to clarify this objective with a little exercise.

Exercise on "Problem Oriented" vs "Solution Focused"

6. What is the significance of being aware of these two approaches when debriefing? How does this relate to your discussions?

<u>Chains of thoughts</u>

7. In some video clips one student gets a lot of attention. The student gets several concrete follow-up questions, and the facilitator also poses related questions to the group. How does this work?
8. Do you think there is any risks that students may feel exposed when receiving this line of questions?

<u>Formulations (or "what works" and "the challenging")</u>

9. You asked about "strategies" and how the students "succeeded". How does this work?
10. You also used another way of questioning: "what are you satisfied with?" How does this work?
11. Earlier you discussed the expression "challenges" e.g., "what challenges did you face?". What do you think of this expression?

<u>Complexity</u>

12. Resilient healthcare maintains that the fact that the healthcare does not succeed in patient safety work may be because healthcare staff have too poor an understanding of what complexity is and because they are not aware that we live in a complex world. I would like you to reflect or discuss what this means
13. What does this mean for the pedagogy of interprofessional simulation-based education?

14. Students seems to blame themselves for not having full control from the get-go. Perhaps this is because they are not aware of complexity being a reality (in the scenario). If so, what can be done in debriefings to pay attention to this?

E. THE NEXT ACTION PLAN

F. SUMMING UP

Focus Group Guide Meeting 5

A. WELCOME

B. AGENDA

Old topics

"Succeeded"
"Strategies"
"Challenges"
Intended learning objectives, Application phase
Observers
Chains of thought

New topics

Complexity, adaptation

C. C. OPENING QUESTION

1. Do you have any reflections since last time?

D. D. IN-DEPTH QUESTIONS

2. What are interprofessional competencies in your opinion?

Show table of intended learning objectives for course and each scenario

3. How do you make use of the learning objectives and how does it work writing them on the whiteboard in the application phase?
4. How do you use the expressions "succeed", "strategies", "challenges" and "contributed"?
5. How do you use video clips?
    1. How do you introduce clips?
    2. How do you follow up?

Inform about findings in Elin's article

    3. How do you ensure that you look for and register behaviours that concern learning objectives, when sitting in the control room?
    4. Discuss looking for CRM-related observable behaviours
6. How did you work with the "complex" and "adaptation"?
7. In relation to video clips of debriefings, discuss the significance of these contradictions:
    1. The situation in a scenario IS messy, and the group succeeds in managing the case vs the students themselves think they are no good because "it was messy"
    2. Asking for rules and instruction vs being able to reflect on variation and adaptation
    3. Focus on shortcomings and the learning of the ideal correct action vs the ability to adapt/deviate in the situation
8. Regarding "Chains of thoughts": How does the persistent questioning to elicit reflection around specific challenges work?

E. THE NEXT ACTION PLAN

F. SUMMING UP

Focus Group Guide Meeting 6

A. WELCOME

B. RECAPITULATE

1. Review of the previous action plan
2. Agenda for the meeting

C. OPENING QUESTION

3. What are your impressions and thoughts after the debriefings?

D. IN-DEPTH QUESTIONS

4. What is the response, when using the word "contributed"?
5. Regarding observers: What is the difference between giving the observers a task looking at a specific student vs looking at a CRM theme
6. Regarding using video: What is the difference between turning on and off video still image before and after vs showing still image while discussing the clip
7. Regarding how to use the 10-second pause, how does it work?
8. Earlier you talked about taking 10 seconds to think about learnings in the application phase. In this clip 10 seconds to think about feelings was used. How does that work?
9. Regarding complexity:

Possibly tell about Manser's "adaptive Coordination"

   1. Do you think talk of “messiness” and “complacency” etc. is expressions of complexity?
   2. Here is a video clip where the words "complexity" and "adjusting" are used. What is going on here?
   3. Do you see any patterns, areas, themes or situations regarding the complex?
   4. Is "heedfulness" a useful concept?

E. CONCLUDING QUESTIONS

10. Any questions or comments?

F. SUMMING UP
